# Human Adenovirus 7d Strains Associated with Influenza-Like Illness, New York, USA, 2017–2019

**DOI:** 10.3201/eid2605.200116

**Published:** 2020-05

**Authors:** Daryl M. Lamson, Adriana Kajon, Michael Popowich, Meghan Fuschino, Kirsten St. George

**Affiliations:** New York State Department of Health, Albany, New York, USA (D.M. Lamson, M. Popowich, M. Fuschino, K. St. George);; Lovelace Respiratory Research Institute, Albuquerque, New Mexico, USA (A. Kajon)

**Keywords:** Human adenovirus 7, influenza-like illness, viruses, colleges, New York, United States, genetic diversity, whole-genome sequence, phylogenetic analysis

## Abstract

Human adenovirus 7d is a respiratory pathogen capable of causing acute respiratory disease of variable severity. Phylogenetic analysis of whole-genome sequences of 15 strains isolated from cases of influenza-like-illness during 2017–2019 demonstrated the circulation of 2 distinct clades of genomic variant 7d in colleges in New York, USA.

Human adenovirus genome (HAdV) type 7d (HAdV-7d) was first detected in the United States in December 2013 in Oregon, in association with acute respiratory disease (ARD) requiring hospitalization (*1*). In 2014, it was detected in more persons with ARD in Oregon and in Illinois in 2 adults with severe pneumonia (*2*). Circulation of this genome type, probably imported from East Asia, where its reemergence was first documented in 2009 (*3*), has been detected since 2013 in other locations and settings in the United States in association with ARD of variable severity in children and adults, including in a long-term care facility in New Jersey (*4*); the US Marine Corps Officer Candidates School in Quantico, Virginia (*5*); and the University of Maryland (https://www.cnn.com/2018/11/21/health/university-of-maryland-death-adenovirus).

The recent increased detection of this distinct genomic variant of HAdV-7 in the United States and its association with severe disease manifestations has prompted public health laboratories to be more vigilant about detection of HAdVs in association with ARD. The Wadsworth Center of the New York State Department of Health (Albany, NY, USA), in collaboration with the Lovelace Respiratory Research Institute (Albuquerque, NM, USA), has monitored the prevalence of respiratory HAdVs detected in New York state since 2012. We characterized 15 HAdV-7 strains isolated from influenza virus–negative respiratory specimens collected from students with influenza-like illness at colleges in Tompkins, Albany, and Clinton counties in New York during the 2016–17, 2017–18, and 2018–19 influenza seasons. We used next-generation whole-genome sequencing and phylogenetic analysis to investigate possible epidemiologic connections among the New York college outbreaks and to monitor the dispersion of this reemerging variant within the United States.

Clinical specimens were initially tested for influenza viruses using a real-time reverse transcription PCR diagnostic panel and subsequently tested for HAdV as previously described (*6*). Samples testing positive for HAdV were processed for molecular typing by amplification and sequencing of hypervariable regions 1–6 of the hexon gene (*7*) and for virus isolation by conventional culture with standard techniques. Intracellular genomic HAdV DNA was purified from infected A549 cells exhibiting cytopathic effect and used for initial genetic characterization by restriction enzyme analysis and for next-generation sequencing with Illumina MiSeq (Illumina, https://www.illumina.com), as previously described (*6*). We aligned genomic sequences generated in this study and reference sequences from GenBank in Geneious Pro R11 using MAFFT (https://www.geneious.com). We constructed a maximum-likelihood tree using MEGA6 (*8*). We also generated in silico digests of the genomic sequences in Geneious Pro R11. We annotated all sequences using VAPiD (*9*) and uploaded to GenBank (accession nos. MH921831–42, MK405661, and MN638755–56).

Initial digestion of viral DNA with endonucleases *Bam*HI, BstEII, *Hpa*I and subsequent in silico digestion of the corresponding complete genomic sequences identified all isolated strains as corresponding to genome type 7d (data not shown). Phylogenetic analysis of whole-genome sequencing demonstrated the co-circulation of 2 distinct clades of HAdV-7d strains in New York, even within the same county, in the sampled time period (Figure). The first clade comprised 11 strains isolated during March 2017–February 2019. Nine of these were genetically related to strains isolated in 2016–2017 in New Jersey (NJ/5644/2016 and NJ/6295/2017), and 2 were more closely related to a 2014 strain isolated in Oregon (OR/CDC2014012.949/2014). The second clade comprised 4 strains isolated during March and April 2017 that were closely related to strain DG01 isolated in 2011 in China and genetically similar to a sample isolated in 2017 from Virginia (VA/5677/2017). Both clades share a common ancestor, strain GZ6965, isolated in Guangdong, China, in 2011.

**Figure:**
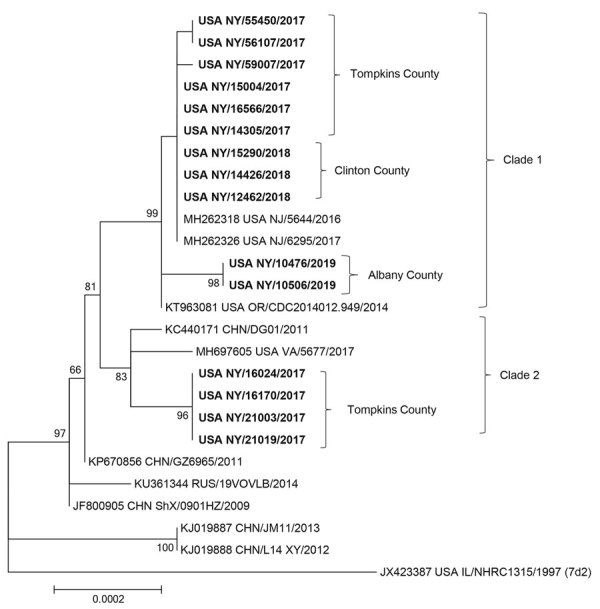
Maximum-likelihood tree showing the phylogenetic relationships of human adenovirus genome type 7d isolates from New York, USA (bold), and reference sequences obtained from GenBank. Accession numbers for reference strains are provided next to the strain designation, country, and year of isolation. Strain USA IL/NHRC1315/1997 of genomic variant 7d2 was included in the analysis as a representative of genome types circulating in the United States before 2013. Nodes with bootstrap values >70 are displayed. Scale bar indicates nucleotide substitutions per site.

Our phylogenetic analysis provides strong evidence of >2 introductions of genomic variant 7d into New York state. Spatiotemporal analysis of a larger genomic sequence dataset with better representation of strains isolated in other US states, as well as in other countries, over an extended period is necessary to more accurately track the introduction of lineages and follow their dispersion.

As in our previous studies and those of others (*4*–*6*,*10*), these findings highlight the importance of HAdV as a causative agent of ARD in civilian communities and the value of college student populations for sentinel surveillance of HAdV activity. These data also provide another example of the power of whole-genome sequencing analysis for the epidemiologic investigation of HAdV-associated disease (*9*,*10*).

All HAdV-7d strains examined in this study were isolated from persons with influenza-like illness. Numerous recent publications report severe and even fatal cases of ARD in association with this genomic variant. All HAdV-7d strains sequenced thus far are indistinguishable by restriction enzyme analysis but not identical when examined at the whole-genome level. Characterization of more strains of diverse origins and associated disease and thorough mining of sequence data is needed to identify candidate determinants of virulence for HAdV-7. Host-related and other environmental risk factors are likely to contribute to the level of susceptibility, clinical presentation, and outcome of the associated disease.
